# Supplementary material for: Nutrient and biomass dynamics for dual-organ yield in turmeric (Curcuma longa L.)
Source: PeerJ. 2025 Aug 28;13:e19933. doi: 10.7717/peerj.19933 (PMC12399080; doi:10.7717/peerj.19933)
Supplement: Supplemental Information 2 [file peerj-13-19933-s002.docx]

**Table 1. Dry matter accumulation of turmeric plant（‾*x*±*s*, *n*=3, g/plant）**

| Sampling Time | Leaf | Stem | Rhizome | Root | Tuber | All plant |
| --- | --- | --- | --- | --- | --- | --- |
| 55 DAP | 2.41±0.13 F/b | 1.56±0.13 E/c | 0.78±0.03 G/d | 0.26±0.04 F/e | - | 5.00±0.20 G/a |
| 69 DAP | 3.56±0.35 F/b | 2.12±0.16 E/c | 1.03±0.03 G/d | 0.49±0.04 F/e | - | 7.19±0.58 G/a |
| 83 DAP | 7.99±0.42 D/b | 4.11±0.28 E/c | 2.36±0.08 F/d | 1.27±0.02 E/e | - | 15.72±0.80 F/a |
| 111 DAP | 10.20±0.22 AB/b | 5.17±0.56 D/c | 7.02±0.24 E/c | 1.67±0.36 D/d | 0.44±0.04 D/e | 24.50±1.41 E/a |
| 125 DAP | 10.56±0.17 A/c | 10.81±0.47 B/c | 13.54±0.08 D/b | 2.42±0.04 BC/d | 2.45±0.20 D/d | 39.77±2.21 D/a |
| 139 DAP | 8.14±0.75 CD/c | 12.96±0.54 AB/b | 20.48±0.64 C/b | 3.28±0.01 A/d | 6.31±0.32 C/c | 51.17±2.32 C/a |
| 167 DAP | 7.33±0.65 D/d | 12.02±0.70 A/c | 24.86±0.94 B/b | 2.73±0.15 B/e | 11.28±0.08 B/c | 58.23±3.17 AB/a |
| 195 DAP | 6.52±0.23 E/d | 9.61±0.36 C/c | 27.14±0.72 A/b | 2.52±0.08 C/e | 14.03±0.25 A/c | 59.81±4.23 A/a |
| 209 DAP | 5.82±0.45 E/d | 8.50±0.48 CD/d | 27.03±0.67 A/b | 2.53±0.04 C/e | 14.70±0.42 A/c | 58.57±3.25 AB/a |

**Table 2. Nitrogen content of turmeric plant（‾*x*±*s*, *n*=3, %）**

| Sampling Time | Leaf | Stem | Rhizome | Root | Tuber |
| --- | --- | --- | --- | --- | --- |
| 55 DAP | 1.721±0.024 B/d | 1.587±0.075 B/c | 2.236±0.163 A/ab | 1.401±0.088 C/c | - |
| 69 DAP | 1.749±0.027 B/d | 1.675±0.053 B/c | 2.047±0.128 A/bc | 1.412±0.066 C/c | - |
| 83 DAP | 2.153±0.068 A/c | 2.077±0.065 A/b | 1.992±0.125 A/c | 1.617±0.025 B/b | - |
| 111 DAP | 2.065±0.097 B/c | 2.545±0.160 A/a | 2.001±0.134 B/c | 1.938±0.122 B/a | 1.521±0.072 C/a |
| 125 DAP | 2.874±0.090 A/a | 2.969±0.175 A/a | 1.654±0.026 B/d | 0.777±0.012 C/d | 1.329±0.021 D/b |
| 139 DAP | 2.700±0.127 A/ab | 2.018±0.063 A/b | 2.150±0.101 A/abc | 1.862±0.146 A/a | 1.318±0.103 B/b |
| 167 DAP | 2.654±0.042 A/b | 2.137±0.067 B/b | 2.337±0.183 AB/a | 1.855±0.141 C/a | 1.027±0.032 D/d |
| 195 DAP | 2.065±0.065 A/c | 2.301±0.144 A/ab | 2.211±0.069 A/ab | 1.534±0.024 B/bc | 1.097±0.034 C/c |
| 209 DAP | 1.607±0.076 B/d | 2.373±0.141 A/ab | 2.222±0.139 A/ab | 1.479±0.023 B/c | 1.065±0.030 C/cd |

**Table 3. Nitrogen accumulation of turmeric plant（‾*x*±*s*, *n*=3, mg/plant）**

| Sampling Time | Leaf | Stem | Rhizome | Root | Tuber |
| --- | --- | --- | --- | --- | --- |
| 55 DAP | 41.46±0.65 A/f | 24.57±1.16 B/e | 17.47±1.37 B/g | 3.58±0.11 C/f | - |
| 69 DAP | 62.24±3.90 A/ef | 35.64±2.36 B/e | 20.99±0.33 C/g | 6.86±0.11 D/f | - |
| 83 DAP | 172.13±5.40 A/c | 85.27±4.01 B/d | 47.01±2.95 C/f | 20.45±0.96 D/d | - |
| 111 DAP | 210.61±9.91 A/b | 131.57±5.14 B/c | 140.61±2.21 B/e | 32.58±2.55 C/c | 6.62±0.21 D/d |
| 125 DAP | 303.59±23.80 A/a | 320.05±18.25 A/a | 223.98±17.56 B/d | 18.77±1.18 C/de | 32.48±1.53 C/c |
| 139 DAP | 220.41±17.28 B/b | 261.53±16.20 B/b | 440.10±20.70 A/b | 60.93±2.87 C/a | 83.21±5.22 C/b |
| 167 DAP | 194.47±3.05 C/bc | 256.92±16.11 C/b | 580.88±36.43 A/a | 50.70±3.97 D/b | 115.84±1.82 D/ab |
| 195 DAP | 134.37±10.53 D/d | 221.23±13.88 C/b | 599.75±28.21 A/a | 38.71±3.03 E/c | 153.87±7.24 D/a |
| 209 DAP | 93.26±2.92 D/e | 201.73±11.81 C/bc | 600.00±37.63 A/a | 37.37±2.93 D/c | 156.46±9.81 C/a |

**Table 4. Phosphorus content of turmeric plant（‾*x*±*s*, *n*=3, %）**

| Sampling Time | Leaf | Stem | Rhizome | Root | Tuber |
| --- | --- | --- | --- | --- | --- |
| 55 DAP | 0.521±0.033 BC/c | 0.608±0.029 AB/bc | 0.650±0.041 A/ab | 0.503±0.039 C/c | - |
| 69 DAP | 0.471±0.022 B/c | 0.570±0.036 A/c | 0.536±0.034 A/c | 0.438±0.027 B/d | - |
| 83 DAP | 0.671±0.011 A/b | 0.656±0.021 A/b | 0.494±0.031 B/c | 0.594±0.028 C/b | - |
| 111 DAP | 0.497±0.027 A/c | 0.667±0.042 AB/b | 0.606±0.037 B/bc | 0.582±0.032 AB/b | 0.700±0.022 B/a |
| 125 DAP | 0.669±0.010 A/b | 0.657±0.041 A/b | 0.702±0.055 A/a | 0.574±0.036 B/b | 0.617±0.039 B/a |
| 139 DAP | 0.758±0.036 A/a | 0.575±0.009 B/c | 0.548±0.043 B/c | 0.644±0.054 B/b | 0.683±0.031 A/a |
| 167 DAP | 0.661±0.010 C/b | 0.779±0.049 A/a | 0.711±0.045 C/a | 0.784±0.061 AB/a | 0.503±0.024 D/b |
| 195 DAP | 0.552±0.009 A/c | 0.604±0.038 A/bc | 0.710±0.052 A/a | 0.416±0.013 B/d | 0.244±0.004 B/c |
| 209 DAP | 0.474±0.007 B/c | 0.521±0.041 D/c | 0.700±0.055 A/a | 0.363±0.006 E/e | 0.238±0.011 C/c |

**Table 5. Phosphorus accumulation of turmeric plant（‾*x*±*s*, *n*=3, mg/plant）**

| Sampling Time | Leaf | Stem | Rhizome | Root | Tuber |
| --- | --- | --- | --- | --- | --- |
| 55 DAP | 12.55±0.59 A/e | 9.45±0.43 A/d | 5.07±0.32 B/e | 1.28±0.06 C/d | - |
| 69 DAP | 16.76±0.79 A/e | 12.10±0.76 B/d | 5.50±0.09 C/e | 2.13±0.07 D/d | - |
| 83 DAP | 53.64±1.68 A/b | 26.93±1.15 B/c | 11.65±0.18 C/d | 7.51±0.35 C/c | - |
| 111 DAP | 50.73±1.54 A/bc | 34.51±2.16 B/c | 42.56±3.34 AB/c | 9.71±0.46 C/c | 3.05±0.05 C/b |
| 125 DAP | 70.62±2.11 B/a | 70.98±4.45 B/b | 95.06±1.49 A/b | 13.87±0.65 C/b | 15.11±0.71 C/b |
| 139 DAP | 61.70±1.86 B/ab | 74.52±5.17 B/b | 112.28±3.52 A/b | 21.08±0.83 C/a | 43.09±0.68 B/a |
| 167 DAP | 48.51±1.04 C/c | 93.67±6.94 B/a | 176.61±8.31 A/a | 21.43±0.87 D/a | 56.75±2.67 C/a |
| 195 DAP | 35.95±0.96 C/d | 57.95±3.16 C/b | 192.69±9.06 A/a | 10.49±0.56 D/c | 34.15±1.07 C/a |
| 209 DAP | 27.56±0.43 D/d | 44.27±2.69 C/bc | 189.28±2.97 A/a | 9.16±0.38 E/c | 34.96±2.74 CD/a |

**Table 6. Potassium content of turmeric plant（‾*x*±*s*, *n*=3, %）**

| Sampling Time | Leaf | Stem | Rhizome | Root | Tuber |
| --- | --- | --- | --- | --- | --- |
| 55 DAP | 3.182±0.095 C/ab | 6.432±0.403 A/a | 4.267±0.268 B/a | 2.986±0.047 C/d | - |
| 69 DAP | 2.875±0.077 B/b | 5.187±0.325 A/b | 3.310±0.104 B/b | 2.357±0.148 B/e | - |
| 83 DAP | 2.845±0.089 B/b | 4.344±0.136 A/c | 3.656±0.287 C/ab | 2.598±0.081 C/e | - |
| 111 DAP | 2.847±0.089 C/b | 3.887±0.183 B/c | 2.173±0.034 D/d | 4.247±0.133 A/a | 3.925±0.362 B/a |
| 125 DAP | 2.669±0.167 B/b | 2.563±0.121 B/d | 2.276±0.178 C/d | 3.819±0.060 A/b | 3.604±0.257 C/a |
| 139 DAP | 3.405±0.267 B/a | 3.242±0.102 B/c | 2.109±0.165 B/d | 5.170±0.381 A/a | 3.070±0.241 B/b |
| 167 DAP | 2.121±0.066 D/c | 2.154±0.104 BC/d | 1.823±0.102 B/d | 4.198±0.197 A/a | 2.125±0.167 C/c |
| 195 DAP | 1.854±0.145 A/c | 2.044±0.128 B/d | 1.538±0.096 A/e | 3.674±0.258 C/bc | 1.721±0.136 C/c |
| 209 DAP | 1.835±0.086 AB/c | 2.023±0.063 B/d | 1.495±0.023 A/e | 3.320±0.260 C/cd | 1.651±0.129 C/c |

**Table 7. Potassium accumulation of turmeric plant（‾*x*±*s*, *n*=3, mg/plant）**

| Sampling Time | Leaf | Stem | Rhizome | Root | Tuber |
| --- | --- | --- | --- | --- | --- |
| 55 DAP | 76.69±1.20 B/d | 99.98±1.57 A/e | 33.29±2.09 C/e | 7.61±0.12 D/f | - |
| 69 DAP | 102.01±1.60 A/d | 110.01±2.17 A/e | 33.95±1.07 B/e | 11.45±0.33 C/f | - |
| 83 DAP | 227.40±3.57 A/b | 178.31±3.39 B/d | 86.26±4.06 C/d | 32.87±1.55 D/e | - |
| 111 DAP | 290.40±4.55 A/a | 201.03±5.30 B/d | 152.51±6.57 C/c | 70.87±3.33 D/d | 17.08±0.80 E/c |
| 125 DAP | 281.81±4.42 B/a | 277.05±6.34 B/b | 308.19±9.16 A/b | 92.24±5.79 C/bc | 88.30±3.75 C/b |
| 139 DAP | 277.40±17.40 C/a | 420.48±10.96 A/a | 431.61±13.74 A/a | 169.19±13.26 D/a | 193.72±7.15 D/a |
| 167 DAP | 155.12±12.16 D/c | 259.37±6.27 B/c | 453.11±14.10 A/a | 114.67±7.80 E/b | 239.74±7.04 BC/a |
| 195 DAP | 120.80±3.79 D/d | 196.37±3.68 C/d | 417.42±12.18 A/a | 92.59±5.81 D/bc | 241.40±7.57 B/a |
| 209 DAP | 106.56±6.68 D/d | 171.90±3.09 C/d | 404.15±11.67 A/a | 83.80±4.57 D/cd | 242.69±7.22 B/a |

**Table 8. Calcium content of turmeric plant（‾*x*±*s*, *n*=3, g/kg）**

| Sampling Time | Leaf | Stem | Rhizome | Root | Tuber |
| --- | --- | --- | --- | --- | --- |
| 55 DAP | 6.43±0.12 A/d | 1.83±0.09 C/e | 1.65±0.05 C/b | 4.64±0.28 B/abc | - |
| 69 DAP | 6.57±0.10 A/d | 1.99±0.03 C/e | 1.86±0.06 C/b | 4.28±0.27 B/bcd | - |
| 83 DAP | 6.45±0.40 A/d | 2.82±0.22 B/d | 1.99±0.13 B/b | 3.44±0.16 B/cd | - |
| 111 DAP | 6.14±0.11 A/d | 6.06±0.48 A/c | 0.93±0.06 C/c | 4.90±0.31 B/ab | 0.33±0.02 C/b |
| 125 DAP | 13.10±0.82 A/c | 8.24±0.65 B/b | 0.90±0.06 D/c | 5.28±0.41 C/a | 0.38±0.03 D/ab |
| 139 DAP | 13.89±0.94 A/c | 7.40±0.46 B/bc | 0.52±0.02 D/d | 4.44±0.32 C/abcd | 0.40±0.01 D/a |
| 167 DAP | 20.12±0.63 A/b | 6.74±0.42 B/c | 0.44±0.02 D/d | 4.24±0.33 C/bcd | 0.08±0.01 D/c |
| 195 DAP | 25.74±2.02 A/a | 16.52±0.26 B/a | 0.15±0.00 D/e | 3.25±0.13 C/d | 0.06±0.00 D/c |
| 209 DAP | 27.50±1.29 A/a | 18.29±0.86 B/a | 0.06±0.00 D/e | 2.56±0.04 C/d | 0.05±0.00 D/c |

**Table 9. Calcium accumulation of turmeric plant（‾*x*±*s*, *n*=3, mg/plant）**

| Sampling Time | Leaf | Stem | Rhizome | Root | Tuber |
| --- | --- | --- | --- | --- | --- |
| 55 DAP | 15.50±1.19 A/e | 2.84±0.18 B/f | 1.29±0.05 B/d | 1.18±0.04 B/f | - |
| 69 DAP | 23.39±1.83 A/e | 4.23±0.20 B/f | 1.91±0.09 B/d | 2.07±0.10 B/f | - |
| 83 DAP | 51.57±4.74 A/d | 11.58±0.37 B/e | 4.69±0.29 B/c | 4.35±0.14 B/e | - |
| 111 DAP | 62.63±4.92 A/d | 31.33±0.98 B/d | 6.51±0.51 C/c | 8.16±0.36 C/cd | 0.15±0.01 D/d |
| 125 DAP | 138.29±8.67 A/b | 89.12±4.19 B/b | 12.15±0.57 C/a | 12.76±0.40 C/b | 0.93±0.04 D/ab |
| 139 DAP | 113.12±7.48 A/c | 95.88±4.01 A/b | 10.53±0.50 B/b | 14.54±0.46 B/a | 2.50±0.16 B/a |
| 167 DAP | 147.20±7.31 A/ab | 80.97±4.03 B/c | 10.82±0.51 C/ab | 11.58±0.55 C/b | 0.91±0.04 C/bc |
| 195 DAP | 167.61±10.51 A/a | 158.74±5.96 A/a | 4.15±0.13 B/c | 8.18±0.39 B/cd | 0.83±0.03 B/c |
| 209 DAP | 159.75±8.95 A/a | 155.45±4.88 A/a | 1.65±0.08 B/d | 6.45±0.20 B/d | 0.67±0.02 B/c |

**Table 10. Magnesium content of turmeric plant（‾*x*±*s*, *n*=3, g/kg）**

| Sampling Time | Leaf | Stem | Rhizome | Root | Tuber |
| --- | --- | --- | --- | --- | --- |
| 55 DAP | 3.45±0.11 C/c | 3.70±0.12 C/c | 6.45±0.30 B/a | 8.87±0.28 A/a | - |
| 69 DAP | 3.31±0.05 C/c | 3.84±0.18 C/c | 6.23±0.39 B/a | 8.38±0.13 A/a | - |
| 83 DAP | 3.17±0.10 C/c | 5.86±0.18 A/b | 4.40±0.35 B/b | 6.19±0.11 A/b | - |
| 111 DAP | 1.67±0.02 D/e | 8.70±0.55 A/a | 3.29±0.21 C/c | 5.78±0.27 B/b | 1.73±0.14 D/a |
| 125 DAP | 3.58±0.22 B/c | 8.35±0.43 A/a | 2.93±0.23 BC/c | 4.08±0.32 B/c | 1.63±0.05 C/a |
| 139 DAP | 4.20±0.07 B/b | 7.37±0.23 A/a | 2.78±0.22 C/c | 4.23±0.07 B/c | 1.60±0.08 C/a |
| 167 DAP | 5.75±0.09 A/a | 6.05±0.10 A/b | 2.29±0.07 C/d | 3.75±0.18 B/c | 1.65±0.05 C/a |
| 195 DAP | 2.25±0.01 B/d | 3.50±0.17 A/c | 1.90±0.12 BC/d | 2.06±0.13 BC/d | 0.92±0.06 C/b |
| 209 DAP | 1.72±0.07 C/e | 3.15±0.05 A/c | 1.79±0.08 C/d | 1.85±0.06 C/d | 0.89±0.04 D/b |

**Table 11. Magnesium accumulation of turmeric plant（‾*x*±*s*, *n*=3, mg/plant）**

| Sampling Time | Leaf | Stem | Rhizome | Root | Tuber |
| --- | --- | --- | --- | --- | --- |
| 55 DAP | 8.31±0.53 A/e | 5.75±0.36 B/f | 5.03±0.08 B/e | 2.26±0.04 C/e | - |
| 69 DAP | 11.80±0.74 A/de | 8.15±0.38 B/f | 6.39±0.10 B/e | 4.06±0.32 C/de | - |
| 83 DAP | 25.36±1.99 A/c | 24.07±0.76 A/d | 10.37±0.81 B/d | 7.83±0.65 B/c | - |
| 111 DAP | 17.00±0.93 C/d | 44.99±2.12 A/c | 23.05±1.85 B/c | 9.62±0.75 C/bc | 0.75±0.01 D/c |
| 125 DAP | 37.77±2.96 C/b | 90.22±4.24 A/a | 39.70±3.11 C/b | 9.85±0.77 D/bc | 3.99±0.15 D/c |
| 139 DAP | 34.29±2.76 C/b | 95.54±4.53 A/a | 56.89±3.57 B/a | 13.85±1.43 D/a | 10.06±0.32 D/b |
| 167 DAP | 42.11±3.32 B/a | 72.70±3.14 A/b | 56.97±3.56 AB/a | 10.24±0.80 C/b | 18.57±0.50 C/a |
| 195 DAP | 14.62±0.92 C/d | 33.62±1.05 B/d | 51.61±2.43 A/a | 5.20±0.36 D/d | 12.90±0.41 C/ab |
| 209 DAP | 9.98±0.78 D/e | 26.78±1.68 B/d | 48.28±2.79 A/a | 4.66±0.37 D/de | 13.01±0.40 C/ab |

**Table 12. Iron content of turmeric plant（‾*x*±*s*, *n*=3,mg/kg）**

| Sampling Time | Leaf | Stem | Rhizome | Root | Tuber |
| --- | --- | --- | --- | --- | --- |
| 55 DAP | 177.85±13.94 C/d | 98.82±6.20 D/d | 258.97±8.12 B/a | 561.47±8.80 A/cd | - |
| 69 DAP | 163.50±12.82 C/d | 103.02±6.46 D/d | 239.86±15.04 B/a | 524.85±32.92 A/d | - |
| 83 DAP | 62.68±3.93 D/e | 125.13±5.89 C/cd | 180.95±5.67 B/b | 478.34±30.00 A/d | - |
| 111 DAP | 50.23±0.79 CD/e | 144.65±2.27 B/c | 20.46±0.96 D/c | 914.39±43.01 A/a | 48.26±2.27 CD/b |
| 125 DAP | 140.00±6.59 B/d | 114.70±8.99 B/d | 4.42±0.07 D/d | 682.15±53.48 A/ab | 53.41±1.68 C/b |
| 139 DAP | 195.86±14.23 B/cd | 90.22±5.66 C/d | 3.21±0.20 D/d | 657.52±20.62 A/abc | 197.57±5.34 B/a |
| 167 DAP | 633.00±29.77 A/b | 54.62±3.43 D/d | 2.58±0.20 D/d | 554.84±34.80 AB/cd | 108.05±3.24 C/b |
| 195 DAP | 1041.54±21.33 A/a | 797.45±62.52 B/a | 1.25±0.06 D/d | 222.84±10.48 C/e | 9.47±0.45 D/c |
| 209 DAP | 1121.86±17.59 A/a | 830.26±26.04 B/a | 0.63±0.03 D/d | 196.69±3.08 C/e | 7.52±0.35 D/c |

**Table 13. Iron accumulation of turmeric plant（‾*x*±*s*, *n*=3, mg/plant）**

| Sampling Time | Leaf | Stem | Rhizome | Root | Tuber |
| --- | --- | --- | --- | --- | --- |
| 55 DAP | 0.43±0.01 A/c | 0.15±0.00 C/d | 0.20±0.02 B/c | 0.14±0.00 C/e | - |
| 69 DAP | 0.58±0.03 A/c | 0.22±0.01 B/d | 0.25±0.02 B/c | 0.26±0.02 B/e | - |
| 83 DAP | 0.50±0.02 A/c | 0.51±0.01 A/c | 0.43±0.03 A/a | 0.61±0.05 A/c | - |
| 111 DAP | 0.51±0.03 B/c | 0.75±0.02 B/c | 0.14±0.01 C/d | 1.52±0.07 A/b | 0.02±0.00 D/c |
| 125 DAP | 1.48±0.67 A/b | 1.24±0.04 A/b | 0.06±0.00 B/e | 1.65±0.09 A/ab | 0.13±0.01 B/b |
| 139 DAP | 1.60±0.73 AB/b | 1.17±0.09 B/b | 0.07±0.00 C/e | 2.15±0.10 A/a | 1.25±0.08 B/a |
| 167 DAP | 4.63±0.15 A/a | 0.66±0.03 C/c | 0.07±0.00 D/e | 1.52±0.07 B/b | 1.22±0.06 B/a |
| 195 DAP | 6.78±0.22 A/a | 7.66±0.60 A/a | 0.03±0.00 C/e | 0.56±0.01 B/d | 0.13±0.01 C/b |
| 209 DAP | 6.52±0.20 A/a | 7.06±0.11 A/a | 0.02±0.00 C/e | 0.50±0.03 B/d | 0.11±0.00 C/b |

**Table 14. Manganese content of turmeric plant（‾*x*±*s*, *n*=3,mg/kg）**

| Sampling Time | Leaf | Stem | Rhizome | Root | Tuber |
| --- | --- | --- | --- | --- | --- |
| 55 DAP | 25.23±1.19 B/c | 14.32±0.90 C/c | 27.26±2.14 B/a | 45.49±1.43 A/a | - |
| 69 DAP | 23.56±1.48 B/c | 13.02±0.82 C/c | 25.68±1.45 B/a | 41.77±1.29 A/a | - |
| 83 DAP | 19.08±0.30 B/d | 5.45±0.09 D/d | 22.52±0.71 B/a | 39.68±2.49 A/a | - |
| 111 DAP | 13.33±0.63 B/e | 9.74±0.31 BC/c | 12.77±0.80 B/b | 31.42±1.48 A/b | 5.40±0.42 C/a |
| 125 DAP | 27.94±2.19 A/c | 8.43±0.26 C/d | 11.39±0.18 C/b | 28.48±0.45 A/bc | 1.81±0.11 D/b |
| 139 DAP | 30.29±0.48 A/b | 7.66±0.11 C/d | 6.66±0.52 C/c | 31.75±1.49 A/b | 1.45±0.11 C/bc |
| 167 DAP | 31.99±0.52 A/b | 7.79±0.12 C/d | 4.51±0.21 C/c | 29.59±2.32 A/bc | 1.42±0.09 C/bc |
| 195 DAP | 47.73±2.99 A/a | 26.52±0.42 B/a | 2.95±0.14 D/d | 15.60±0.83 C/d | 0.36±0.01 D/c |
| 209 DAP | 50.31±2.37 A/a | 29.36±1.38 B/a | 1.07±0.02 D/d | 13.39±0.84 C/d | 0.25±0.01 D/c |

**Table 15. Manganese accumulation of turmeric plant（‾*x*±*s*, *n*=3, mg/plant）**

| Sampling Time | Leaf | Stem | Rhizome | Root | Tuber |
| --- | --- | --- | --- | --- | --- |
| 55 DAP | 0.06±0.01 A/d | 0.02±0.00 B/e | 0.02±0.00 B/e | 0.01±0.00 B/e | - |
| 69 DAP | 0.08±0.00 A/d | 0.03±0.00 B/e | 0.03±0.00 B/de | 0.02±0.00 B/de | - |
| 83 DAP | 0.15±0.01 A/c | 0.02±0.00 C/e | 0.05±0.00 B/d | 0.05±0.00 B/c | - |
| 111 DAP | 0.14±0.01 A/c | 0.05±0.00 B/d | 0.09±0.00 A/b | 0.05±0.00 B/c | 0.00±0.00 C/c |
| 125 DAP | 0.30±0.01 A/a | 0.09±0.01 C/b | 0.15±0.01 B/a | 0.07±0.01 C/b | 0.01±0.00 D/b |
| 139 DAP | 0.25±0.01 A/b | 0.10±0.01 B/b | 0.14±0.00 B/a | 0.10±0.01 B/a | 0.01±0.00 C/b |
| 167 DAP | 0.23±0.01 A/b | 0.09±0.01 BC/b | 0.11±0.00 B/b | 0.08±0.01 C/b | 0.02±0.00 D/a |
| 195 DAP | 0.31±0.02 A/a | 0.26±0.01 A/a | 0.08±0.00 B/c | 0.04±0.00 C/cd | 0.01±0.00 C/b |
| 209 DAP | 0.29±0.01 A/a | 0.25±0.01 A/a | 0.03±0.00 B/de | 0.03±0.00 B/d | 0.00±0.00 B/c |

**Table 16. Zinc content of turmeric plant（‾*x*±*s*, *n*=3,mg/kg）**

| Sampling Time | Leaf | Stem | Rhizome | Root | Tuber |
| --- | --- | --- | --- | --- | --- |
| 55 DAP | 8.13±0.53 C/d | 25.65±2.01 B/c | 42.22±3.31 A/a | 26.58±2.08 B/c | - |
| 69 DAP | 8.94±0.65 C/d | 23.91±0.38 B/c | 39.46±2.25 A/a | 29.14±1.88 B/bc | - |
| 83 DAP | 10.13±0.79 C/d | 17.74±0.56 C/d | 36.17±2.27 A/a | 32.92±2.07 AB/ab | - |
| 111 DAP | 6.52±0.41 C/d | 24.23±0.76 A/c | 25.59±1.20 A/c | 24.89±0.39 A/c | 12.23±0.58 B/a |
| 125 DAP | 12.03±0.75 C/c | 35.63±1.68 A/b | 24.28±1.14 B/c | 27.40±1.29 B/c | 9.98±0.31 C/a |
| 139 DAP | 16.54±1.04 BC/bc | 27.28±1.02 AB/c | 16.64±1.29 BC/d | 36.34±1.71 A/a | 7.31±0.23 C/b |
| 167 DAP | 19.97±1.25 B/b | 18.95±0.62 B/d | 15.63±1.23 B/d | 34.66±2.72 A/a | 2.35±0.18 C/c |
| 195 DAP | 30.35±2.38 B/a | 51.05±1.60 A/a | 14.50±0.46 C/d | 20.32±0.64 C/d | 1.07±0.08 D/d |
| 209 DAP | 35.02±1.65 B/a | 57.84±0.91 A/a | 13.22±0.21 C/d | 17.30±1.09 C/d | 0.64±0.04 D/d |

**Table 17. Zinc accumulation of turmeric plant（‾*x*±*s*, *n*=3, mg/plant）**

| Sampling Time | Leaf | Stem | Rhizome | Root | Tuber |
| --- | --- | --- | --- | --- | --- |
| 55 DAP | 0.02±0.00 B/e | 0.04±0.00 A/f | 0.03±0.00 A/f | 0.01±0.00 C/e | - |
| 69 DAP | 0.03±0.00 B/e | 0.05±0.00 A/f | 0.04±0.00 AB/f | 0.01±0.00 C/e | - |
| 83 DAP | 0.08±0.01 A/d | 0.07±0.00 A/e | 0.09±0.00 A/e | 0.04±0.00 B/d | - |
| 111 DAP | 0.07±0.00 C/d | 0.13±0.01 B/d | 0.18±0.01 A/d | 0.04±0.00 D/d | 0.01±0.00 E/e |
| 125 DAP | 0.13±0.01 C/c | 0.39±0.03 A/b | 0.33±0.02 AB/b | 0.07±0.00 D/c | 0.03±0.00 D/c |
| 139 DAP | 0.14±0.01 C/c | 0.35±0.02 A/b | 0.34±0.03 A/b | 0.12±0.00 C/a | 0.05±0.00 D/a |
| 167 DAP | 0.15±0.01 C/b | 0.23±0.02 B/c | 0.39±0.03 A/a | 0.10±0.00 C/b | 0.03±0.00 D/c |
| 195 DAP | 0.20±0.01 B/a | 0.49±0.03 A/a | 0.39±0.04 A/a | 0.05±0.00 C/c | 0.02±0.00 C/d |
| 209 DAP | 0.20±0.01 B/a | 0.49±0.03 A/a | 0.36±0.03 A/ab | 0.04±0.00 C/d | 0.01±0.00 C/e |

**Table 18. Copper content of turmeric plant（‾*x*±*s*, *n*=3,mg/kg）**

| Sampling Time | Leaf | Stem | Rhizome | Root | Tuber |
| --- | --- | --- | --- | --- | --- |
| 55 DAP | 1.29±0.02 B/e | 1.86±0.15 B/d | 1.65±0.08 B/e | 13.47±0.85 A/b | - |
| 69 DAP | 1.94±0.06 C/de | 2.03±0.16 C/cd | 3.43±0.27 B/c | 14.90±1.17 A/a | - |
| 83 DAP | 2.58±0.16 C/cd | 2.69±0.04 C/c | 10.38±0.84 B/a | 19.57±1.23 A/a | - |
| 111 DAP | 3.86±0.30 B/b | 1.94±0.09 C/d | 4.88±0.12 B/b | 14.78±0.70 A/a | 1.80±0.08 C/c |
| 125 DAP | 2.91±0.09 BC/c | 2.11±0.13 C/cd | 4.11±0.13 B/b | 11.99±0.19 A/b | 2.20±0.10 C/b |
| 139 DAP | 2.90±0.23 B/c | 2.57±0.08 B/c | 4.03±0.19 B/b | 10.16±0.16 A/c | 3.23±0.14 B/a |
| 167 DAP | 6.24±0.25 B/a | 3.88±0.12 C/b | 3.73±0.12 C/c | 12.69±1.00 A/b | 2.49±0.12 C/b |
| 195 DAP | 5.01±0.08 B/ab | 7.74±0.12 A/a | 3.39±0.27 C/cd | 7.15±0.45 A/c | 1.69±0.08 C/c |
| 209 DAP | 4.70±0.07 B/b | 8.67±0.54 A/a | 3.17±0.20 C/d | 6.41±0.50 AB/c | 1.55±0.05 C/c |

**Table 19 Copper accumulation of turmeric plant（‾*x*±*s*, *n*=3, mg/plant）**

| Sampling Time | Leaf | Stem | Rhizome | Root | Tuber |
| --- | --- | --- | --- | --- | --- |
| 55 DAP | 0.003±0.00 A/f | 0.003±0.00 A/e | 0.001±0.00 B/f | 0.004±0.00 A/e | - |
| 69 DAP | 0.007±0.00 A/e | 0.005±0.00 A/e | 0.004±0.00 A/f | 0.007±0.00 A/e | - |
| 83 DAP | 0.021±0.00 A/d | 0.011±0.00 B/d | 0.025±0.00 A/d | 0.025±0.00 A/b | - |
| 111 DAP | 0.039±0.00 A/b | 0.010±0.00 C/d | 0.034±0.00 A/c | 0.025±0.00 B/b | 0.001±0.00 D/e |
| 125 DAP | 0.031±0.00 B/c | 0.023±0.00 C/c | 0.056±0.00 A/b | 0.029±0.00 BC/b | 0.006±0.00 D/d |
| 139 DAP | 0.024±0.00 C/d | 0.033±0.00 BC/b | 0.083±0.00 A/a | 0.034±0.00 B/a | 0.021±0.00 C/b |
| 167 DAP | 0.046±0.00 B/a | 0.047±0.00 B/b | 0.093±0.00 A/a | 0.035±0.00 B/a | 0.028±0.00 B/a |
| 195 DAP | 0.033±0.00 B/c | 0.074±0.01 A/a | 0.092±0.00 A/a | 0.018±0.00 C/c | 0.024±0.00 BC/ab |
| 209 DAP | 0.028±0.00 B/c | 0.074±0.01 A/a | 0.086±0.00 A/a | 0.016±0.00 C/d | 0.023±0.00 BC/ab |
